# Factors that determine the motivation to change alcohol consumption in cardiovascular patients: an exploratory study

**DOI:** 10.1186/1940-0640-8-S1-A39

**Published:** 2013-09-04

**Authors:** Myrna Keurhorst, Angelien Sieben, Gerard Schippers, Miranda Laurant, Bas Bredie

**Affiliations:** 1 Radboud University Nijmegen Medical Center, Scientific Institute for Quality of Healthcare, Nijmegen, NL

## Background

Cardiovascular diseases (CVDs) remain the primary cause of mortality worldwide. Harmful use of alcohol is an important causative lifestyle risk factor for CVD. Motivation to change one's lifestyle is an important determinant of actually changing lifestyle. However, little is known about factors that could be used to modify an individual's motivation to change.

## Aim

To examine in patients with manifest CVD which factors determine motivation to change alcohol consumption.

## Participants

2037 subsequent patients in the departments of General Internal Medicine, Cardiology, Neurology, and Vascular Surgery, most of whom had had a recent CVD event, participated in the study.

## Methods

They completed a cardiovascular risk assessment, including a computerized self-report lifestyle questionnaire and motivation to change behavior. In this presentation we focus, however, on alcohol use.

## Results

On a scale from 1 (not willing to change) to 5 (definitely willing to change), patients scored 1.68 willingness to change their alcohol consumption. Being female, older and a smoker were positively associated with the motivation to change. The relationship between participants' age and their motivation to reduce their alcohol consumption was moderated by their sex. These factors together, however, explained only 2.5% of the total variance. Although being female, older and a smoker were associated with stronger motivation to change their alcohol consumption to a healthy norm, also other individual patient factors appeared to be involved. Based on this, personalized medicine and motivational interviewing, which could help choosing the right strategy for fostering changes in lifestyle, may be more effective.

